# Hoxb1 Controls Anteroposterior Identity of Vestibular Projection Neurons

**DOI:** 10.1371/journal.pone.0034762

**Published:** 2012-04-02

**Authors:** Yiju Chen, Masumi Takano-Maruyama, Bernd Fritzsch, Gary O. Gaufo

**Affiliations:** 1 Department of Biology, University of Texas at San Antonio, San Antonio, Texas, United States of America; 2 Department of Biology, University of Iowa, Iowa City, Iowa, United States of America; Tokyo Medical and Dental University, Japan

## Abstract

The vestibular nuclear complex (VNC) consists of a collection of sensory relay nuclei that integrates and relays information essential for coordination of eye movements, balance, and posture. Spanning the majority of the hindbrain alar plate, the rhombomere (r) origin and projection pattern of the VNC have been characterized in descriptive works using neuroanatomical tracing. However, neither the molecular identity nor developmental regulation of individual nucleus of the VNC has been determined. To begin to address this issue, we found that Hoxb1 is required for the anterior-posterior (AP) identity of precursors that contribute to the lateral vestibular nucleus (LVN). Using a gene-targeted Hoxb1-GFP reporter in the mouse, we show that the LVN precursors originate exclusively from r4 and project to the spinal cord in the stereotypic pattern of the lateral vestibulospinal tract that provides input into spinal motoneurons driving extensor muscles of the limb. The r4-derived LVN precursors express the transcription factors Phox2a and Lbx1, and the glutamatergic marker Vglut2, which together defines them as dB2 neurons. Loss of Hoxb1 function does not alter the glutamatergic phenotype of dB2 neurons, but alters their stereotyped spinal cord projection. Moreover, at the expense of Phox2a, the glutamatergic determinants Lmx1b and Tlx3 were ectopically expressed by dB2 neurons. Our study suggests that the Hox genes determine the AP identity and diversity of vestibular precursors, including their output target, by coordinating the expression of neurotransmitter determinant and target selection properties along the AP axis.

## Introduction

The developing hindbrain is organized into an orthogonal coordinate system from which different functional neuronal columns originate along the dorsoventral (DV) axis that span single or multiple rhombomeres along the anteroposterior (AP) axis [1], [2]. In the dorsal hindbrain, the functional diversity of sensory columns or nuclei is determined by signals along the DV axis, whereas longitudinally identity is determined by signals along the AP axis [3], [4], [5], [6], [7]. That latter mechanism ensures that sensory nuclei are in register with peripheral sensory input (auditory or vestibular; [8], [9]), and the output of the sensory nuclei is matched with central targets along the AP axis [6]. The majority of hindbrain sensory nuclei have been defined through the expression and genetic fate map of genes that encode, among others, four basic helix-loop-helix (bHLH) and homeodomain (HD) proteins [4], [5], [7], [10], [11], [12], [13], [14], [15], [16], [17]. These proteins are expressed at specific coordinates along the DV axis. For example, Atoh1-expressing precursors of the cochlear nucleus are born in the dorsal-most cellular domain of the alar plate, whereas Phox2b- and Lbx1-expressing precursors of the solitary tract and trigeminal sensory nuclei, respectively, are born in progressively more ventral progenitor domains [3], [5], [7], [11], [12], [14], [16], [17]. Dorsal sensory neurons can be further categorized by their expression of specific HD proteins and whether they are dependent (dA1–dA4 neurons) or independent (dB1–dB4 neurons) on roof plate signals [4], [5], [7], [18]. For example, auditory, visceral, and somatosensory neurons are classified as dA1, dA3, and dB1–dB4 neurons, respectively [5], [7], [11], [12], [16], [17]. The functional classification of dorsal sensory neurons stems from genetic ablation studies in which elimination of the roof plate resulted in the loss of dA1–dA4 neurons, while maintaining Lbx1-expressing dB1–dB4 neurons [5], [7].

In addition to defining the various dorsal neuronal subtypes, some HD proteins are essential for determining the neurotransmitter phenotypes of sensory neurons [3], [14], [15], [16], [19], [20], [21]. The HD proteins Phox2b, Lhx1/5, and Tlx3, for example, not only define dA3, dA4/dB1/dB4, and dB3 neurons, but they also determine the noradrenergic, GABAergic, and glutamatergic fate of these neurons, respectively. This molecular classification has been used to define the precursors for every sensory nuclei except for those of the vestibular nuclear complex (VNC), which collectively are involved in regulating eye movement, balance coordination, and posture [22], [23].

Compared to the DV axis, less is known about the mechanisms that regulate the columnar identity of sensory nuclei along the AP axis. However, genetic fate mapping and retrograde labeling studies have provided insight into the rhombomere (r) contribution to sensory columns in the developing hindbrain [6], [11], [24]. Genetic fate map of r2 and r3/r5, for example, show that the subdivisions of the vestibular nuclei can be segregated along the AP axis based on their rhombomeric origin [24]. This finding is complemented by retrograde labeling studies that show that the lateral vestibulospinal tract (LVST) originate predominantly from rhombomere (r4), whereas projections of the vestibuloocular system receive contributions from multiple rhombomeres [24], [25], [26], [27], [28], [29], [30], [31], [32], [33]. These findings suggest that sensory columns are composed of modules derived from specific rhombomeres that are assembled along the AP axis into individual nuclei. Support for this hypothesis comes from loss of function studies of the Hox gene encoding transcription factors, the major AP determinants of the hindbrain and spinal cord [6], [34], [35], [36], [37], [38], [39]. Loss of Hoxb1 and the combination of Hoxa3 and Hoxb3, for example, leads to misspecification of dA3 noradrenergic neurons of the rostral part of the solitary tract nucleus derived from r4 and r5, respectively [3] whereas conditional deletion of Atoh1 in r3 and 5 leads to loss of specific parts of cochlear nucleus neurons [40]. Hox genes have also been shown to be necessary for the specification and columnar organization of the cochlear and trigeminal principal sensory nuclei [6], [41]. Like most sensory relay nuclei, the individual components of the VNC are likely to be under Hox gene regulation.

**Figure 1:**
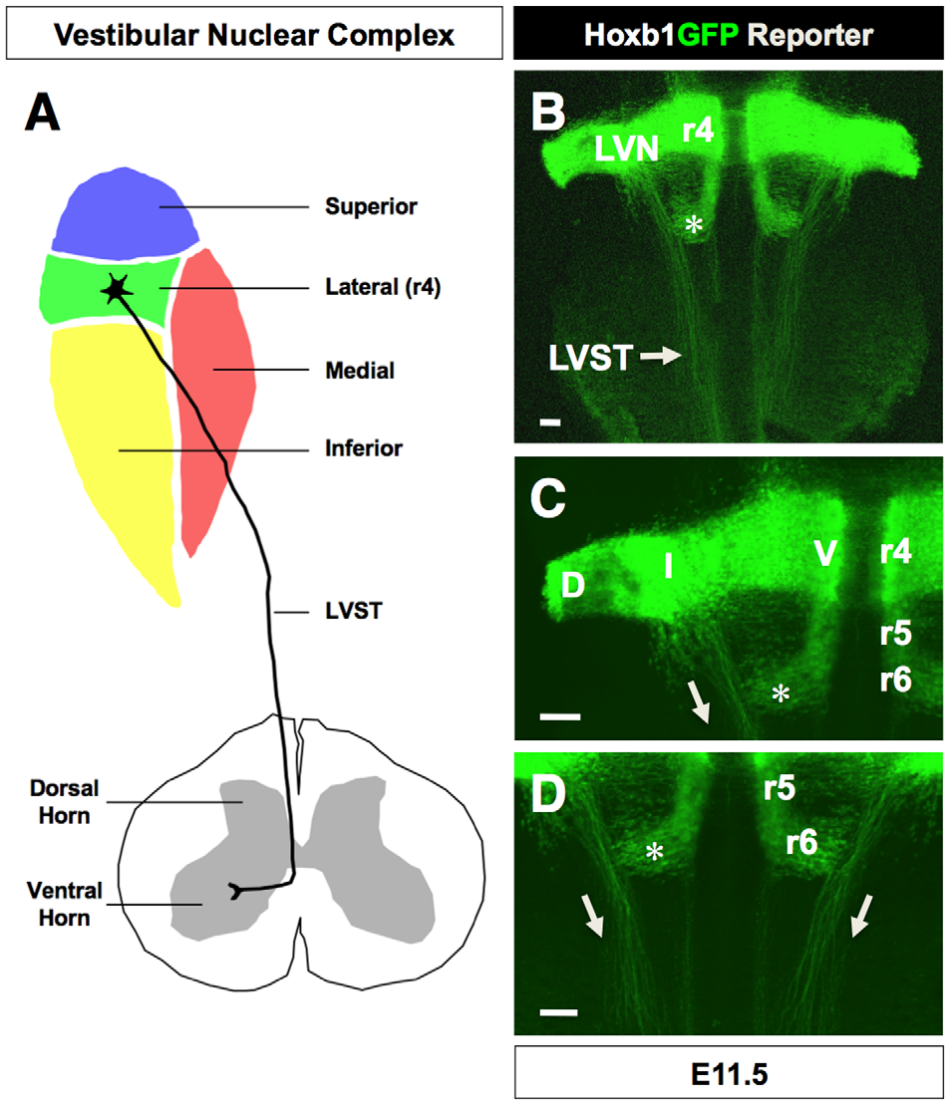
Hoxb1-GFP reporter defines the rhombomere 4 origin of the lateral vestibulospinal tract. (**A**) Schematic diagram of the vestibular nuclear complex (VNC) and the projection pattern of the lateral vestibulospinal (LVST) originating from the lateral vestibular nucleus (LVN). The LVST extends the spinal cord, ends on motor neurons activating extensors and is responsible for decerebration rigidity. The medial VST, which also projects ipsilaterally (not shown for clarity), ends at the thoracic level and is responsible for head eye movement integration. (**B, C, D**) Flat mount preparation of E11.5 embryo harboring the GFP gene targeted into the Hoxb1 locus. The pial surface of the embryo is exposed to better visualize Hoxb1-GFP-labeled axons of the LVST originating from rhombomere 4 (r4) and projecting to the ipsilaterally spinal cord (arrow). The LVST lies dorsolateral to the facial branchial motoneurons that have migrated caudolaterally from r4 to r6 (asterisk). Abbreviations: D, dorsal; I, intermediate; V, ventral; r, rhombomere. Scale bars (B–D = 100 µm).

Hoxb1 is unique among the Hox genes in its dynamic and restricted expression pattern in the developing hindbrain [38], [42], [43], [44], [45], [46]. Hoxb1 is initially expressed throughout the neuroepithelium from the prospective r4 region to the caudal hindbrain and spinal cord. By midgestation (embryonic day 9.5, E9.5), the expression of Hoxb1 becomes restricted to progenitors and early postmitotic cells of r4 as a consequence of a conserved autoregulatory mechanism [44], [47]. Various vertebrate species show that the expression of Hoxb1 is coincident with the r4 origin of precursors of the LVN [9], [25], [27], [28], [31], [48] (Fig. 1A). These observations suggest that Hoxb1 may be required for the AP identity of the LVN. In this study, we show using a Hoxb1-GFP reporter that the pattern of axonal projections of a population of r4-derived neurons is consistent with those previously observed for the LVN [24], [26], [27], [31], [32], [33], [48]. In the absence of Hoxb1 we observed that the projection pattern of these r4-derived neurons was lost, and that their identity was transformed into a more anterior r2/r3-like identity. These findings suggest that the projection patterns and molecular identities of sensory neurons that contribute to the VNC complex may be under homeotic control along the AP axis.

## Materials and Methods

### Animals

The Hoxb1-GFP reporter and Hoxb1 null mouse lines used for this study have been previously reported and approved by IACUC protocol #MU041 [38]. Appropriate mouse lines were bred to obtain embryos at various stages of development. The day of plug was marked as E0.5. Embryos were harvested at E10.5, E11.5, E12.5, E13.5 and E15.5. Briefly, pregnant female mice were euthanized by CO_2_ asphyxiation. Embryos were removed from uteri and immediately placed in cold phosphate buffered saline (PBS) and transferred to cold 4% formaldehyde/PBS for fixation. Embryos were fixed for 30–60 minutes (depending on age), washed several times in cold PBS, and subsequently underwent a sucrose gradient of 10%, 20%, and 30% sucrose/PBS for cryoprotection. Cryoprotected embryos were imbedded in OCT medium filled molds and stored at −80°C for storage. For histological analysis, adult control and Hoxb1^−/−^ mice were euthanized by CO_2_ asphyxiation and perfused with cold PBS followed by 4% formaldehyde/PBS. The brains were extracted and post-fixed overnight, washed in PBS, and processed for paraffin imbedding.

### Immunohistochemistry and tract tracing

Frozen embryos were sectioned with a cryostat (Microm HM550) in either the transverse or horizontal plane at 12–20 µm intervals and mounted on charged glass slides (20 µm transverse sections were used for quantitative analysis). Briefly, mounted tissue sections were washed in PBS/01% Triton-100 (PBST), blocked in 5% normal goat serum/milk (NM) PBST for 1 hour, washed in PBST, and incubated in 2.5% NM-PBST with primary antibodies overnight. The following day, tissues were washed in PBST, incubated in 2.5% NM-PBST with appropriate secondary antibodies for 1 hour, and washed in PBST. All solutions and steps were kept at 4°C. An anti-fading solution (Vectashield, Vector) was added to immunolabeled tissue sections prior to visualization and acquisition by confocal microscopy (Pascal 5 or LSM 500, Zeiss). The following primary antibodies were used for this study: rabbit anti-GFP (1∶1000, Molecular Probes); rabbit anti-Phox2b (1∶1000, generated in the Gaufo lab); rabbit anti-Phox2a (1∶1000, generated in the Gaufo lab); guinea pig anti-Lbx1 (1∶13,000; provided by T. Müller); guinea pig anti-Lmx1b (1∶13,000, provided by T. Müller); mouse anti-Lhx1/5 (1∶100, Developmental Hybridoma); mouse anti-Gad1 (1∶100, Developmental Hybridoma); rabbit anti-Tlx3 (1∶13,000, provided by T. Müller); mouse anti-Ascl1 (1∶100, provided by D. Anderson); guinea pig anti-Vglut2 (1∶5000, Chemicon). The following host-specific secondary antibodies were used for this study: Alexa-Fluor 488 (1∶1000, Molecular Probes); Alexa-Fluor 594 (1∶1000, Molecular Probes); and Alexa-Fluor 645 (1∶1000, Molecular Probes).

Tract tracing was performed using NeuroVue maroon and NeuroVue red filter stripes as previously described [49]. Filter strips soaked with these dyes were inserted into the rostral spinal cord and labeled fibers and imaged using flattened whole mount preparations of the neural tube. In addition, small pieces of hair soaked in Neurovue dyes were inserted under visual control with an 488 nm epifluorescent equipped dissecting scope (Leica M250) into the GFP labeled r4 of Hoxb1-GFP heterozygote and homozygote embryos to anterogradely fill the LVN axons. Proper positioning in the mediodorsal axis was verified through the labeling of the vestibular afferents.

## Results

### Hoxb1-GFP reporter defines r4 origin of projection neurons to the ipsilateral spinal cord

To address our working hypothesis that Hoxb1 is required for early LVN patterning, we re-examined the origin of the LVN in mouse embryos harboring a green fluorescent protein (GFP) reporter targeted into the Hoxb1 locus (Hoxb1-GFP) [38]. Exposure of the pial surface of hindbrain flatmount preparations of E11.5 Hoxb1-GFP embryos showed two prominent axonal tracts originating from the intermediate region of r4 and projecting ventromedially to the ipsilateral spinal cord (Fig. 1B–D). This pattern is consistent with retrograde labeling experiments in the chick embryo showing that the LVN gives rise to ipsilateral projecting lateral vestibulospinal tract (LVST) [24], [26], [27], [28], [29], [30], [31], [32], [33]. It should be noted that these descending Hoxb1-GFP-positive axons are distinct from those derived from cochlear, viscerosensory, and somatosensory neurons, whose axons project as ascending tracts predominantly to the thalamus [23], [50]. The LVST is also distinguishable from the more medial reticulospinal tract and medial longitudinal fasciculus [22], [23]. Based on the r4 origin and projection pattern of Hoxb1-GFP-positive axons that is similar to the LVST, we hypothesize that the r4 determinant Hoxb1 is required for its r4 or AP identity. It should be noted that it is possible that a population of neurons contributing to the LVN may also originate from neighboring rhombomeres [24]. Previous retrograde labeling experiments have shown that although the majority of neurons were labeled in r4, a small set of neurons was also labeled in r3 and r5. Nevertheless, our finding suggests that many precursors contributing to the LVN originate from r4.

### Projection pattern of the lateral vestibulospinal tract is lost in Hoxb1 mutant embryos

**Figure 2:**
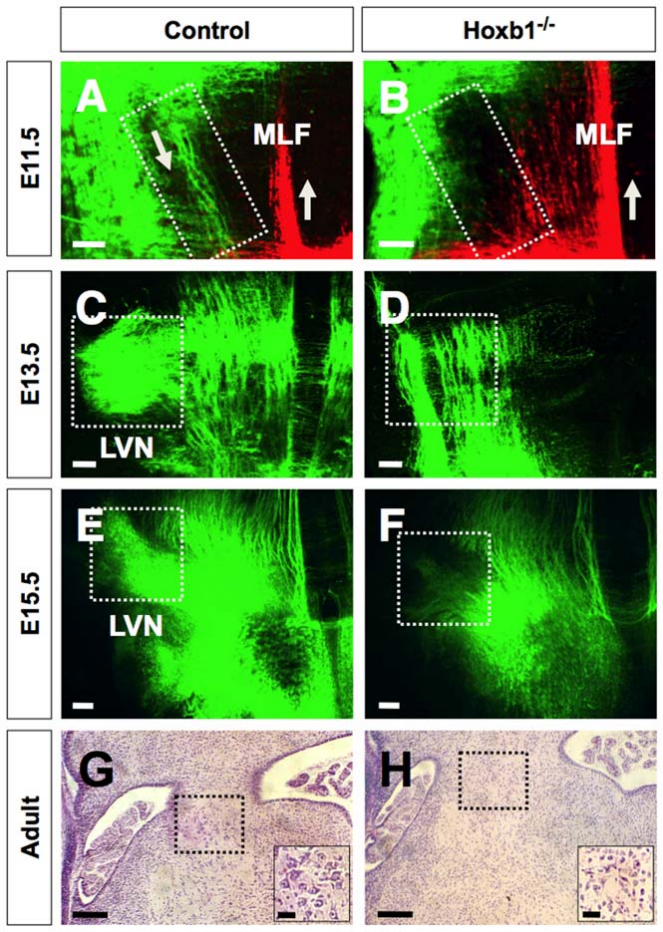
The projection pattern of the lateral vestibulospinal tract is missing Hoxb1^−/−^ embryos. (**A, B**) Fluorescent anterograde labeling of E11.5 control and Hoxb1^−/−^ embryos injected with NeuroVue maroon (green) in the intermediate region of r4 and NeuroView (red) on the medial side of the caudal hindbrain to label the medial longitudinal fasciculus (MLF). Note prominent labeling of the axons emanating from the intermediate region of r4 is absent in the Hoxb1^−/−^ embryo. (**C, D**) Fluorescent retrograde labeling of E13.5 control and Hoxb1^−/−^ embryos injected with NeuroVue maroon (green) on the ipsilateral side of the spinal cord. Note prominent labeling of the LVN neurons in the control, but absent in the Hoxb1^−/−^ embryo. (**E, F**) Fluorescent retrograde labeling of E15.5 control and Hoxb1^−/−^ embryos injected with NeuroVue maroon (green) on the ipsilateral side of the spinal cord. Note prominent labeling of the LVN neurons in the control, but absent in the Hoxb1^−/−^ embryo. (**G, H**) Transverse section through the lateral vestibular nucleus (LVN) in adult control and Hoxb1^−/−^ hindbrain stained for hematoxylin and eosin. Lower right inset shows high magnification of the outlined area of the LVN. Large multipolar neurons are prominent in the LVN area of the control mouse, whereas only small to medium sized neurons are observed in the LVN area of the Hoxb1^−/−^ mouse. Scale bars (A–F = 100 µm; G, H = 250 µm; G, H insets = 50 µm).

We next tested whether the formation of the LVN is dependent on Hoxb1 by examining the axonal projection pattern in Hoxb1^−/−^ embryos. We examined the projection pattern of the LVN by fluorescent dye labeling because the activity of the Hoxb1-GFP reporter is dependent on a Hoxb1-dependent autoregulatory mechanism, and therefore absent in the Hoxb1^−/−^ embryo [38], [44]. To define the LVN and the associated LVST, we used the fluorescent lipophilic dye NeuroVue maroon (pseudo-colored green) to anterograde label the LVST by applying dye soaked filter strip in the intermediate region of r4 in E11.5 control and Hoxb1^−/−^ embryos (Fig. 2A, B) using the GFP labeling as marker with a fluorescent equipped dissecting scope. We also placed a different fluorescent lipophilic dye NeuroVue red (pseudo-colored red) at the level of the ipsilateral caudal hindbrain at the level of r7 to label the medial longitudinal fasciculus (Fig. 2A, B). The surrounding LVN neurons and their ipsilateral axonal projections can be readily labeled with this approach in the wildtype (Fig. 2A). However, no projections were observed in Hoxb1^−/−^ embryos (Fig. 2B). This result suggests that either the LVN neurons do not form or do not project their axons to the spinal cord at E11.5. Since our injections were limited to r4 we cannot determine if there is any contribution from r3 and r5.

To begin to address the issues of formation of LVN neuron from other rhombomeres or potential delay in r4 origin of LVN maturation and thus formation of LVST axons, we performed complementary retrograde labeling experiments in E13.5 and E15.5 control and Hoxb1^−/−^ embryos. We applied fluorescent dye soaked filter strip (NeuroVue, green) in a broad region of the caudal hindbrain to ensure labeling of the majority of descending axonal tracts emanating from and traversing through the region encompassing the r4-derived LVN. In control E13.5 and E15.5 embryos, retrograde-labeled axons following the trajectory of the LVST could be traced to originate from a large cluster of neurons in the ventrolateral region of the hindbrain (Fig. 2C, E). This appears to be the LVN. In Hoxb1^−/−^ embryos, although many other axons were observed to terminate and traverse the same level of the hindbrain as in control embryos, we failed to label the LVST and see a prominent cluster of neurons in the ventrolateral region normally occupied by the LVN (Fig. 2D, F). Judging from paraffin histology, it appears likely that LVN is at best represented by small interneurons in the adult hindbrain (Fig. 2G, H). Our data suggest that the LVN and its stereotypic LVST is dependent on Hoxb1, but the fate of the LVN or its precursors is unclear beyond the fact that they are not contributing to a LVST. Investigating the expression of activated caspase −3 expression and TUNEL in E10.5–E11.5 embryos did not indicate any elevated level of apoptosis in the dorsal area of r4, suggesting that LVN either never proliferated or was transformed into a different phenotype (data not shown) [3], [38]. Since there is no strong argument in the literature that Hox codes define proliferation rate of a rhombomere we concentrated on the possibility that LVN neurons undergo a cell fate change in the absence of Hoxb1 at the time when LVN neurons are born [51].

### LVN precursors classified as dB2 neuronal subtype via HD protein expression profile

**Figure 3:**
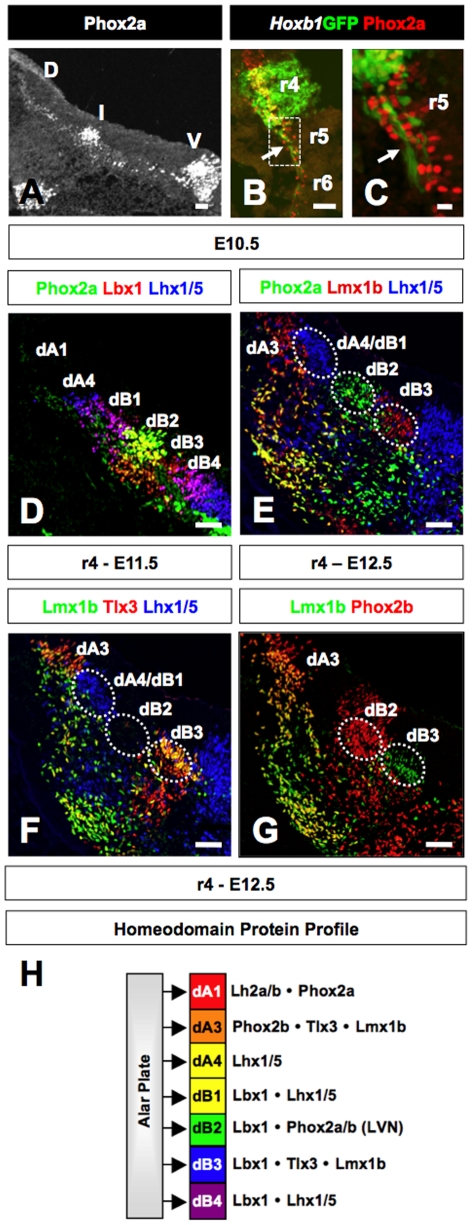
R4-derived projection neurons defined as Phox2a/Lbx1-double positive dB2 neurons. (**A**) Transverse section of E10.5 through r4 of control embryo immunolabeled for Phox2a. The expression of Phox2a in the intermediate (I) region of r4 approximates the area from which the LVST originate (V, ventral; D, dorsal). (**B, C**) Horizontal section of E10.5 embryo harboring a Hoxb1-GFP reporter immunolabeled for GFP (green) and Phox2a (red). Early axon projection from neurons that will contribute to the lateral vestibulospinal tract is indicated by an arrow. Panel C represents magnification of outlined area in panel B. (**D**) Transverse section through r4 of E11.5 control embryo triple-immunolabeled for the transcription factors Phox2a (green), Lbx1 (red), Lhx1/5 (blue). The dB2 neurons are defined by the co-expression (yellow) of Phox2a and Lbx1. (**E**) Transverse section through r4 of E12.5 embryos triple-immunolabeled for the transcription factors Phox2a (green), Lmx1b (red), Lhx1/5 (blue). The Phox2a-single positive dB2 neurons (green) are flanked dorsally by Lhx1/5-single positive dA4/dB1 neurons (blue) and ventrally by Lmx1b-single positive dB3 neurons (red). (F) Transverse sections through r4 of E12.5 embryos triple immunolabeled for the HD proteins Lmx1b, Tlx3, and Lhx1/5. (G) Transverse sections through r4 of E12.5 embryos double immunolabeled for the HD proteins Lmx1b and Phox2b. (H) Summary of immunolabeling experiments showing the seven neuronal subtypes that express different combinations of HD proteins in the dorsal region of r4. The neuronal precursors that contribute to the LVN are defined as dB2 neurons based on the combinatorial expression of Lbx1, Phox2a, and Phox2b. Scale bars (A, B = 50 µm; C = 10 µm; D–G = 50 µm).

To further define the LVN precursors derived from r4 beyond giving rise to the LVST and later determine their fate in the absence of Hoxb1, we screened through a host of HD proteins expressed in the dorsal neural tube [5], [7], [12], [16], [52]. In transverse sections of E10.5 Hoxb1-GFP reporter embryos, we found that one of these HD proteins, Phox2a, was expressed in the putative location of the LVN in intermediate region of r4 (Fig. 3A and see Fig. 1B, C). Longitudinal sectional planes allowed us to visualize at single cell resolution the early descending axonal projections of Hoxb1/Phox2a-expressing precursors (Fig. 3B, C). These findings suggest that the Hoxb1/Phox2a-expressing cells contribute to the LVST and thus are at least part of the LVN. It should be noted that the Phox2a-expressing neurons span multiple rhombomeres, with different levels of expression along the AP axis (Fig. 3B, C; Phox2a^+^ cells in r5; and data not shown). It is possible that like the expression of the transcription factors Atoh1, Phox2b, Lbx1, and Hb9/Isl11 among neurons of the cochlear, solitary tract, trigeminal somatosensory, and motor nuclei, respectively [3], [7], [11], [17], [35], [36], [53], the Phox2a-expressing intermediate column may give rise to functionally similar neurons that span multiple rhombomeres along the AP axis (i.e., components of the LVN).

**Figure 4:**
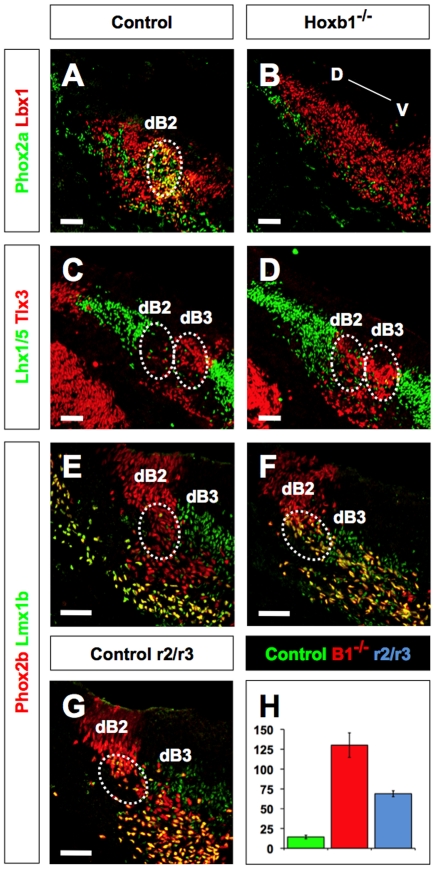
Hoxb1 is required for the anterior-posterior identity of dB2 neurons in r4. (**A, B**) Transverse sections through r4 of E11.5 control and Hoxb1^−/−^ embryos immunolabeled for Phox2a and Lbx1. Phox2a expression is lost or dramatically reduced among dB2 neurons, while Lbx1 is maintained in Hoxb1^−/−^ embryo compared to control. (**C, D**) Transverse sections through r4 of E11.5 control and Hoxb1^−/−^ embryos immunolabeled for Lhx1/5 and Tlx3. dB1 Lhx1/5-positive neurons are juxtaposed to dB2-Tlx3-negative neurons in the control embryo. In the Hoxb1^−/−^ embryo, Tlx3 is ectopically expressed in the dB2 domain (Tlx3-positive dB2 neurons abut Lhx1/5-positive dB1 neurons). (**E–G**) Transverse sections of E11.5 embryos through r4 of control and Hoxb1^−/−^, and r2/r3 of control immunolabeled for Phox2b and Lmx1b. Phox2b and Lmx1b are not co-expressed among control dB2 neurons, whereas co-expression is observed in r4 of Hoxb1^−/−^ and r2/r3 of control embryos. The migration of dB2 neurons in control appears to migrate in the radial direction. In r4 of Hoxb1^−/−^ and r2/r3 of control embryos, dB2 neurons appear to migrate in the ventromedial direction. (**H**) Quantitative analysis of the number of Phox2b/Lmx1b-double positive dB2 neurons in r4 of E11.5 control (green bar, n = 3) and Hoxb1 mutant (B1^−/−^, red bar, n = 3) embryos and r2/r3 of control (r2/r3, blue bar, n = 3) embryos. The number of double-positive dB2 neurons was significantly more in r4 of Hoxb1^−/−^ and r2/r3 of control embryos compared to r4 of control embryos (*P*<0.005). Data given as mean (cells/section) ± standard error mean. Scale bars (A–G = 50 µm).

To understand the relationship of r4-derived LVN precursors with the various classes of sensory neuronal subtypes, we performed a detailed characterization of Phox2a expression in dorsal r4 [3], [4], [5], [7], [10], [11], [12], [13], [15], [16], [17], [19], [20], [21], [52]. To accomplish this goal, we performed double- and triple-immunolabeling experiments in E11.5 and E12.5 wild-type control embryos with antibodies against a variety of HD proteins (Fig. 3D–H). Consistent with previous studies, we identified seven molecularly distinct dorsal neuronal subtypes (dA1, dA3, dA4, dB1, dB2, dB3, and dB4) in r4 [7]. From these experiments, we were able to define the r4-derived LVN precursors as dB2 neurons through the combined expression of Phox2a, Phox2b, and Lbx1. Importantly, these experiments allowed us to define the identity of the r4-derived cell bodies belonging to the Hoxb1-GFP labeled axons that contribute to the LVN (Fig. 1C, D; Fig. 3B, C). However, it should be noted that this column of Phox2a^+^Lbx1^+^Phoxb2b^+^ neurons are contiguous with dB2 domain located in more caudal rhombomeres (i.e., r5 and r6), but are absent in the comparable domain located in more rostral rhombomeres (i.e., r1, r2, and r3 (Fig. S1; Fig. 4; and data not shown).

### Hoxb1 is required for the AP identity of LVN precursors

By defining molecularly the identity of the LVN precursors, we were in a position to ask if the LVN and its associated LVST were lost in Hoxb1^−/−^ embryos. The phenotype associated with the loss of Hox genes is highly conserved and stereotypic, where a segment of the body is usually transformed into a more anterior segment [2], [35], [39], [45], [54], [55], [56]. We therefore predicted that the loss of Hoxb1 in r4 would result in a phenotype characteristic of the more anterior rhombomeres [3]. Because redundancy among Hox genes may occur in r4, the combined loss of Hoxb1, Hoxa2, and Hoxb2 (the Hox genes expressed in r4 at the time of dorsal neuronal patterning) would be required in order for r4 to transform into the non-Hox gene-expressing r1 [3], [57]. However, the loss of one or two Hox genes in r4 should lead to a transformation that is more r2/r3-like. To test this hypothesis, we first analyzed the expression of Phox2a and Lbx1. In control E11.5 embryos, the expression of Phox2a (green) in the dB2 domain is relatively high and is co-expressed with Lbx1, which spans a much broader domain (red, dB1–dB4) (Fig. 4A). In contrast, the expression of Phox2a is dramatically reduced in Hoxb1^−/−^ mutant embryos, whereas the expression of Lbx1 remained intact (note the low expression of Phox2a is masked by the high expression of Lbx1) (Fig. 4B). The fact that the expression of Lbx1 remained intact in the dB2 domain suggested that the loss or reduction in the intensity of Phox2a expression is a consequence of a change in cell identity.

To test this idea, we analyzed the expression of Lhx1/5 and Tlx3 among dB1 and dB3 neurons, respectively. In control E11.5 embryos the expression of Lhx1/5 and Tlx3 precisely bordered within r4 the Phox2a-expresssing dB2 neurons dorsally and ventrally, respectively (Fig. 4C). The Phox2a-expressing dB2 neurons were devoid of Lhx1/5 and Tlx3 expression (compare circles in Fig. 4C). In contrast, in Hoxb1^−/−^ mutant embryos the dB2 domain was completely infiltrated with Tlx3-expressing neurons (Fig. 4D). This phenotype may be a consequence of ectopic production of dB3-like neurons from the dB2 progenitor domain or aberrant migration of db3 neurons into the space normally occupied by Phox2a-expressing dB2 neurons. To address this issue, we analyzed the expression of Phox2b, which labels both progenitors and postmitotic neurons of the dB2 domain [52], and Lmx1b, whose expression coincides with Tlx3 (Fig. 3F). In control E11.5 embryos, the expression of Phox2b perfectly complemented the more ventral Lmx1b-expressing dB3 domain (Fig. 4E). In Hoxb1^−/−^ embryos, the Lmx1b-expressing dB3 domain remained intact (Fig. 4F). However, a population of Lmx1b-expressing cells was observed to emanate from the Phox2b-expressing dB2 domain, as indicated by their co-expression. This finding suggests that the loss of Phox2a expression in the dB2 domain of Hoxb1^−/−^ embryos is a consequence of a change in cell identity rather than abnormal migration of Tlx3/Lmx1b-expressing cells from the dB3 domain.

To test whether the change in cell identity of dB2 neurons in r4 is a consequence of an anterior transformation characteristic of Hox gene loss-of-function, we analyzed the normal expression of Phox2b and Lmx1b in the more anterior rhombomeres (r2/r3). In control E11.5 embryos, the expression of Phox2b and Lmx1b in r2/r3 qualitatively and quantitatively closely approximated the dB2 domain in r4 of Hoxb1^−/−^ embryos (Fig. 4G, H). These observations support the hypothesis that the loss of Hoxb1 results in the transformation of r4 dB2 neurons into r2/r3-like dB2 neurons. We also found that the homologous dB2 domain in r5 was dependent on the combination of Hoxa3 and Hoxb3 (Fig. S1). Together, these findings are consistent with the idea that columnar or nuclear homologues that span the AP axis are under Hox gene regulation [6], [35], [37], [39]. As previously shown, this may occur by convergence of Hox transcription factors directly on regulatory elements that control the expression of DV patterning molecules, such as Phox2b [58]. Shaping the pattern of dB2 neurons along the AP axis may therefore be a manifestation of the Hox transcription factors converging upon DV patterning programs [37], [38].

### Change in Ascl1 expression level associated with anterior transformation of dB2 neurons

**Figure 5:**
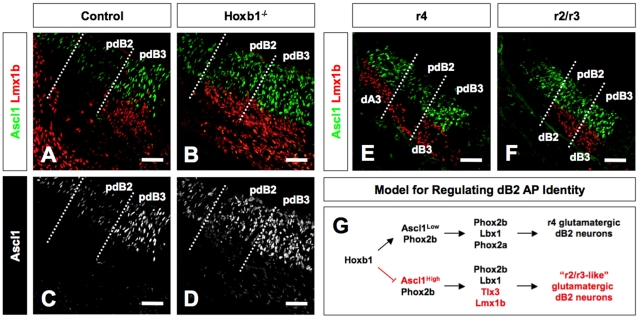
Hoxb1 modulates expression of Ascl1 in the dB2 progenitor domain at the level of r4. (**A–D**) Transverse sections through r4 of E11.5 control and Hoxb1^−/−^ embryos immunolabeled for Ascl1 and Lmx1b. In the control embryo, pdB2 expresses low levels of Ascl1 and no Lmx1b. In contrast, the expression of Ascl1 is increased and Lmx1b is ectopically produced from the pdB2 domain in the Hoxb1^−/−^ embryo. Panels C and D show Ascl1 staining only for clarity. (**E, F**) Transverse section through r4 and r2/r3 of control E11.5 embryo immunolabeled for Ascl1 and Lmx1b. The pdB2 domain in r4 expresses low levels of Ascl1 with no postmitotic Lmx1b neurons differentiating from this domain. In contrast, the high level of Ascl1 expression in the pdB2 domain of r2/r3 is associated with differentiating Lmx1b neurons, an expression profile similar to r4 of Hoxb1^−/−^ embryos. This suggests that in the absence of Hoxb1, the r4 pdB2 domain transforms into the more anterior r2/r3 pdB2 domain. (**G**) A model for regulating the AP identity of dB2 progenitors in r4. The pdB2 domain expresses low levels of Ascl1 and Phox2b (upper pathway). Postmitotic dB2 neurons emerge from the pdB2 domain expressing Phox2b, Lbx1, and Phox2a. As postmitotic dB2 neurons exit the pdB2 domain, they appear to migrate in a radial direction. In the absence of Hoxb1 (lower pathway), the expression of Phox2b within the pdB2 domain and glutamatergic markers among dB2 neurons appears unperturbed, whereas the expression of Ascl1 within the pdB2 domain is dramatically increased. This molecular phenotype is associated with the loss of Phox2a expression among postmitotic dB2 neurons and ectopic expression or de-repression of Tlx3 and Lmx1b from the pdB2. In the absence of Hoxb1, postmitotic dB2 neurons also appear to migrate in the ventromedial direction. These findings show the hallmark of a homeotic or anterior transformation (r4 dB2→r2/r3 dB2). This finding defines one of the earliest processes by which Hox genes regionally control the neurogenic program (Ascl1→Tlx3→glutamatergic neurons). However, it remains to be determined whether Hoxb1 directly regulates this pathway or via an intermediate molecule(s). Scale bars (A–F = 50 µm).

Although we observed ectopic expression of Lmx1b from the Phox2b-expressing dB2 domain in Hoxb1^−/−^ embryos, the expression pattern of Phox2b among progenitors was not observably different from that of control embryos. To further test that that transformation of dB2 neurons in r4 of Hoxb1^−/−^ embryos is a consequence of changes at the progenitor level, we analyzed the expression of the neurogenic bHLH transcription factor Ascl1 in combination with Lmx1b (Fig. 5A–D). We analyzed Ascl1 expression because it is both necessary and sufficient for Lmx1b and Tlx3 expression in mouse and chick embryos, respectively [13], [21]. We reasoned that the ectopic expression of Lmx1b and Tlx3 among Hoxb1-deficient dB2 neurons should be associated with changes in Ascl1 expression in dB2 progenitors (pdB2). In E11.5 control embryos, we observed that Ascl1 was expressed at low levels in a pepper-and-salt-like pattern in the pdB2 domain (Fig. 5A, C). In contrast, Ascl1 was expressed at relatively higher levels in the more ventral pdB3 domain, from which the Lmx1b-expressing dB3 neurons arise. In Hoxb1^−/−^ embryos, we found a uniform increase in the expression of Ascl1 in the pdB2 domain (Fig. 5B, D). The high expression level of Ascl1 in the pdB2 approximated that of the more ventral pdB3 domain. This was associated with the emergence of Lmx1b-expressing neurons from the now high Ascl1-expressing pdB2 domain. Consistent with the idea of an anterior transformation, we observed that expression pattern of Ascl1 at the level of control r2/r3 approximated that of r4 in Hoxb1^−/−^ (Fig. 5E, F). This was also observed in the pdB2 of r5 in Hoxa3^−/−^Hoxb3^−/−^ embryos (Fig. S1). Together, our findings indicate that in Hoxb1^−/−^ embryos, the loss of Phox2a expression and associated ectopic production of Tlx3 and Lmx1b neurons from the pdB2 domain is correlated with an increase in Ascl1 expression. This provides a model in which the AP identity of dB2 neurons or LVN precursors is an indirect consequence of a repressor activity of Hoxb1 at the level of Ascl1-expressing progenitors (Fig. 5G; this flow diagram incorporates the results from the next section).

### Hoxb1 controls the molecular identity of glutamatergic dB2 and dB3 neurons in r4

**Figure 6:**
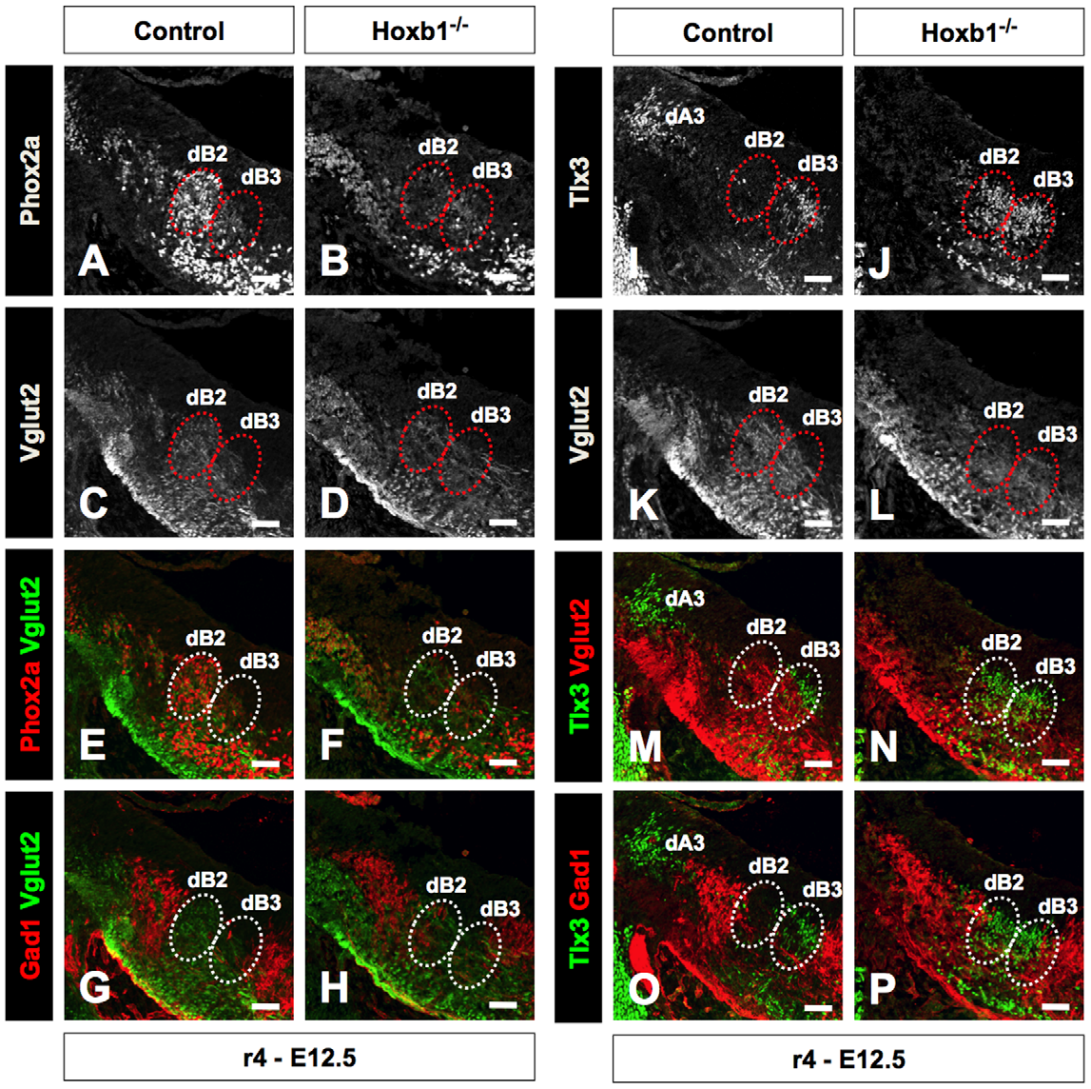
Hoxb1 is required for the glutamatergic identity of dB2 neurons in r4. (**A–F**) Transverse sections through r4 of E12.5 control and Hoxb1^−/−^ embryos immunolabeled for Phox2b and the glutamatergic marker Vglut2. The expression of Phox2a in the dB2 domain is lost or dramatically reduced in the Hoxb1^−/−^ embryo compared to the control (A, B). Vglut2 staining in the cellular domain occupied by dB2 and dB3 neurons is unchanged in the Hoxb1^−/−^ embryo compared to the control embryo (C, D). Panels E and F are composite of images A–D that were separated for clarity. (**G, H**) Transverse sections through r4 of E12.5 control and Hoxb1^−/−^ embryos immunolabeled for VGLUT2 (green) and Gad1 (red). The complementary expression of Vglut2 and Gad1 is unperturbed in the absence of Hoxb1. (**I–M**) Transverse sections through r4 of E12.5 control and Hoxb1^−/−^ embryos immunolabeled for Tlx3 and the glutamatergic marker Vglut2. Tlx3 is ectopically expressed in the dB2 domain in the Hoxb1^−/−^ embryo compared to the control (I, J). Again, the staining for Vglut2 in the cellular domain occupied by dB2 and dB3 neurons is unchanged in and the Hoxb1^−/−^ embryo compared to the control embryo (C, D). Panels M and N are composite of images I–L that were separated for clarity. (**O, P**) Transverse sections through r4 of E12.5 control and Hoxb1^−/−^ embryos immunolabeled for Tlx3 (green) and Gad1 (red). Tlx3 is ectopically expressed in the dB2 domain while the expression of Gad1 remains complementary to this domain in the Hoxb1^−/−^ embryo. This suggests that the ectopic expression of Tlx3 in the dB2 domain does not perturb the neurotransmitter phenotype from this cellular domain. Scale bars (A–P = 50 µm).

The transcription factors Tlx3 and Lmx1b have been shown to be determinants of glutamatergic neurons [16], [19], [20], [21]. It follows that the ectopic production of Tlx3 and Lmx1b in the dB2 domain of Hoxb1^−/−^ embryos should also lead to the ectopic production of glutamatergic neurons. To test this, we analyzed the expression of Phox2a and Tlx3 in combination with the glutamatergic marker Vglut2. We also looked at the expression of the GABAergic marker Gad1. Glutamatergic and GABAergic neurons are normally generated in complementary domains [15], [16], [19], [20], [21]. We confirmed that in the absence of Hoxb1, the expression of Phox2a in the dB2 is lost or dramatically reduced concurrent with the ectopic production of Tlx3 (Fig. 6A, B, I, J). With the ectopic expression of Tlx3 in the dB2 domain, we reasoned that there would be a loss the complementary GABAergic domain in place of glutamatergic neurons. Interestingly, we found that the expression of Vglut2 encompassed both dB2 and dB3 domains, and appeared unaffected in Hoxb1^−/−^ embryos (Fig. 6C, D, K, L; panels E, F, M, and N are composite images of panels A–B and I–L; the channels were separated for clarity). Furthermore, the complementary expression of Vglut2 and Gad1 were maintained in Hoxb1^−/−^ embryos (Fig. 6G, H, O, P). This suggests that although Tlx3 is a known determinant for glutamatergic neurons, it appears that it is not the sole determinant of the glutamatergic pathway – at least not for dB2 neurons. More importantly, these findings suggest the presence of two glutamatergic neuronal populations that can be defined by specific molecular signatures (i.e., Phox2a dB2 versus Tlx3/Lmx1b dB3 neurons), which may account for differences in cellular behavior. This is analogous to the motor neuron system, where cholinergic branchial/visceral and somatic motor neurons are classified based on their molecular expression profile [35], [39], [52], [53].

## Discussion

### Molecular and cellular origin of the LVN

The LVN provides an isolated model to study evolutionarily conserved mechanisms that regulate the modular organization of sensory nuclear columns in the developing hindbrain [59]. The LVN is part of a larger nuclear complex (VNC) that collectively spans the majority of the hindbrain from the cerebellum to the calamus scriptorius [9], [22], [23], [24], [60], [61]. Fate mapping experiments using the quail-chick chimera and the Cre/LoxP reporter system in the mouse have demonstrated that specific nuclear columns of the VNC originate from either individual or multiple rhombomeres [24], [27]. Our study using the Hoxb1-GFP reporter suggests that r4 gives rise to the vast majority of precursors contributing to the LVN and forming the LVST. Previous retrograde labeling in E14.5 mouse embryos showed that although cell bodies of the LVN reside primarily in r4, they are also observed in r3 and r5, as inferred by an r3/r5 Cre/LoxP/RosaLacZ lineage reporter [24]. This observation suggests that the mouse LVN may receive contributions from multiple rhombomeres. Alternatively, the LVN precursors in r3 and r5 may have migrated from r4, a suggestion in line with our data of complete loss of LVST in Hoxb1 null mice. The dramatic loss we report here in the pattern of axons leaving the intermediate region of r4 to the ipsilateral spinal cord is consistent with what has been previously shown in other vertebrates [24], [26], [27], [30], [31], [32], [33], [62], [63]. Whether the remaining nuclear columns of the VNC arise from individual or multiple rhombomeres, requires combining genetic fate mapping, retrograde labeling, and immunolabeling of established dorsal neuronal subtype markers to provide better resolution of the origin and rostrocaudal migratory behavior of precursors of the VNC.

### Contribution of dorsal neuronal subtypes to the lateral vestibular nucleus

Like other sensory nuclei in the hindbrain and spinal cord, the organization of the VNC is likely under the influence of AP and DV pattering programs [2], [18]. The studies herein provide support for the rhombomeric contribution of vestibular projection neurons to the VNC, specifically r4 to the LVN. However, it is less clear whether a single or multiple dorsal neuronal subtypes (dA1–dA4, dB1–dB4) contribute to the LVN along the DV axis. In the present study, we identified LVN precursors as glutamatergic dB2 neurons based on the expression of specific HD proteins and the glutamatergic marker VGLUT2. However, like most nuclei in the VNC, the LVN contains multiple neurotransmitter subtypes, including GABAergic neurons [64]. How then is neurotransmitter phenotype diversity generated within the LVN or other nuclei of the VNC? At least two possibilities may help explain this question. The first possibility suggests that neuronal diversity is generated from a common progenitor pool that produces multiple neuronal subtypes. In our study, for example, the first wave of neurons generated from the pdB2 domain may give rise to glutamatergic neurons, while a second wave produces GABAergic neurons. This scenario has been demonstrated in the generation of excitatory (glutamatergic) and inhibitory (GABAergic) interneurons in the spinal cord [21]. It was shown that Ascl1 is required for the expression of Tlx3 (a glutamatergic determinant) during an early phase of neurogenesis. At a later developmental stage, Ascl1 controls the GABAergic determinant Ptf1a. A single progenitor domain can therefore produce multiple neurotransmitter subtypes at different stages of development. Obtaining multiple neurotransmitter phenotypes within a single sensory nucleus can also be explained through contributions from several dorsal neuronal subtypes. For example, somatosensory neurons of the trigeminal nucleus originate from the Lbx1-expressing cellular domain in the developing hindbrain [7]. The expression of Lbx1 encompasses dB1 to dB4 neurons, where dB1/dB4 and dB2/dB3 are defined by postmitotic molecular determinants of the GABAergic and glutamatergic phenotypes, respectively [7], [19], [20]. Therefore, the complex neurotransmitter composition of the trigeminal nucleus may manifest from the convergence of multiple dorsal neuronal precursors. A similar mechanism may contribute to the neurotransmitter phenotypes in the LVN. Glutamatergic neurons may arise from dB2 neurons, while GABAergic neurons may arise from neighboring dB1 neurons. Whether a single or multiple dorsal neuronal subtypes contribute to individual nuclei of the VNC, future studies may benefit from genetic fate mapping in which the expression of genes that are unique to individual dorsal progenitors or postmitotic neurons are temporally controlled (i.e., tamoxifen-inducible Cre).

### A model for the homeotic control of the vestibular nuclear complex

The columnar organization of the VNC is intriguing with respect to AP patterning and the contribution of the Hox genes. It has been suggested that the VNC encompasses the entire alar plate of the hindbrain [24], including the cerebellum that consists only of an anterior vestibular nucleus with an output pattern similar to the deep cerebellar nucleus in lampreys [59], If this is the case, then r1 or the vestibular sensory neurons contained within r1 (the only rhombomere devoid of Hox gene expression) may represent a Hox gene “ground state” [57]. That is, r1 represents the fundamental molecular and cellular template from which successive action of Hox genes from r2 to the caudal hindbrain determine the different identities of vestibular sensory neurons along the AP axis. For example, while Aoth1 expressing precursors in r2–6 give rise to cochlear nucleus neurons [40], Atoh1 positive neurons in r1 contribute to the cerebellum [10]. At the molecular level, this hypothesis appears to be warranted. For example, loss of Hoxa2, the only Hox gene expressed in r2, causes the complete loss of the HD protein Lbx1 [3]. As in the case for Hox genes, Lbx1 expression is absent in r1. Thus, at the molecular level, Hoxa2-deficient r2 appears to be transformed into r1. In the present study, we show that the loss of Hoxb1 results in a transformation of r4 dB2 neurons into r2/r3-like dB2 neurons. Specifically, we show that the expression of Tlx3 and Lmx1b is ectopically produced from the pdB2 domain. Lbx1-deficient embryos also display this molecular phenotype [7]. These findings suggest that Lbx1 contributes to a Hox gene regulatory network that allows molecular distinction between neighboring neuronal subtypes with a common neurotransmitter phenotype. Interestingly, molecular homologues of dB2 neurons from r2 to the caudal hindbrain appear to occur as repeating units (i.e., r2/r3, r4/r5/r6) like those observed for the Hox-dependent branchial motor system [7], [65]. If the entire dB2 neuronal column from r2 to the caudal hindbrain gives rise to vestibular sensory neurons, then it would suggest that the entire VNC may be under homeotic gene control. The present study provides the first test of this hypothesis, showing that the r4 component of the VNC is under homeotic gene control.

Previous studies showing loss of Hox gene function in the hindbrain have demonstrated a clear association with a homeotic transformation. A homeotic transformation is typically associated with missing or ectopic gene/protein expression and changes in the migratory behavior of neurons that resembles those of more anterior rhombomeres [3], [39], [45], [54]. For example, mutations in Hoxb1 and Hoxa3/b3 respectively in r4 and r5 leads to a complete loss of Phox2b noradrenergic neurons (dA3) in the dorsal hindbrain, a characteristic of r2 and r3, which are normally devoid of Phox2b expression in the alar plate. The loss of Hoxb1 also leads to misspecification of facial branchial motor neurons (FBMs) [38], [39], [42], [45]. In Hoxb1-deficent embryos, r4-derived FBMs, which normally migrate from r4 to the caudal hindbrain, instead migrate ventrolaterally like trigeminal motor neurons in r2 and r3 [39], [45], [54]. Indeed homeotic transformation of rhombomeres and in particular the r4 derived Mauthner cell that projects like the LVN to the spinal cord is one of the best known examples of homeotic transformation using retinoic acid of rostral into caudal rhombomeres [66], [67]. Consistent with a more anterior expression of posterior Hox genes with retinoic acid treatment, our data suggest that even simple deletion of one Hox gene can result in a comparable homeotic transformation of rhombomere specific differentiation. However, loss of function of Hox codes results into more rostral identity.

Although the aforementioned findings provide evidence for a homeotic transformation, insight into a mechanism for how this conserved phenomenon takes place remains poorly understood, largely due to the limited availability of known molecular markers that determine neuronal subtypes and neurotransmitter phenotypes. The present study, however, benefits from the myriad of neuronal determinants that have been meticulously characterized in the dorsal spinal cord and hindbrain [5], [12], [13], [17], [68], [69], [70]. As a consequence, the level of molecular detail that we provide adds new insight into a mechanism contributing to a homeotic transformation. The data presented herein suggest that the transformation of dB2 LVN precursors in r4 may be a consequence of a mechanism by which Hoxb1 regulates the expression levels of the proneural gene Ascl1. The modification of proneural gene expression by different combination of Hox genes from r2 to the caudal hindbrain may determine the different neuronal identities that contribute to the VNC along the AP axis.

## Supporting Information

Figure S1 **Hoxa3 and Hoxb3 are required for the AP identity of dB2 neurons in rhombomere 5.** (**A, B**) Transverse sections through r5 of E11.5 control and Hoxa3^−/−^b3^−/−^ embryos double immunolabeled for the HD proteins Phox2a and Lbx1. In the Hoxa3^−/−^b3^−/−^ embryo, the expression of Lbx1 appears unperturbed whereas Phox2a is dramatically reduced. (**C**) Transverse sections through r2/r3 of E11.5 control embryo double immunolabeled for the HD proteins Phox2a and Lbx1. Phox2a at the intersection of this AP and DV level is expressed, if at all, at very low levels. (**D**) Cell counting of Phox2a^+^Lbx1^+^-positive cells in r5 of control (n = 3) and Hoxa3^−/−^b3^−/−^ (n = 3) embryos, and r2/r3 of control embryos (n = 3). The number of Phox2a^+^Lbx1^+^-positive cells was significantly reduced in Hoxa3^−/−^b3^−/−^ embryos compared to control embryos (*P*<0.05). Few Phox2a^+^Lbx1^+^-positive cells were found in r2/r3 of control embryos. The bar represents standard deviation from the mean. (**E, F**) Transverse sections through r5 of E11.5 control and Hoxa3^−/−^b3^−/−^ embryos double immunolabeled for the bHLH transcription factor Ascl1 and Phox2b. The expression of Lbx1 appears unperturbed in the Hoxa3^−/−^b3^−/−^ embryo, but Phox2a is dramatically reduced. Note that the expression of Ascl1 in the dB2 progenitor domain (pdB2) is relatively high in the Hoxa3^−/−^b3^−/−^ embryo compared to the control. (**G, H**) Transverse sections through r5 of E11.5 control and Hoxa3^−/−^b3^−/−^ embryos double immunolabeled for Ascl1 and Lmx1b. In the control embryo Lmx1b-expressing cells are associated with the dB3 progenitor domain (pdB3). In the Hoxa3^−/−^b3^−/−^ embryo, Lmx1b-expressing cells ectopically associated with the pdB2 domain. (**I, J**) Transverse sections through r5 of E11.5 control and Hoxa3^−/−^b3^−/−^ embryos double immunolabeled for the HD proteins Lmx1b and Phox2b. In the control, the expression of Lmxb1 in the dB3 domain is non-overlapping with the more dorsal Phox2b-expressing dB2 domain. In the Hoxa3^−/−^b3^−/−^ embryo, the Lmx1b-expressing dB3 domain remains intact but an ectopic population of Lmx1b-expressing cells was observed to originate from the Phox2b-expressing pdB2 domain. (**K**) Transverse sections through r2/r3 of E11.5 control embryo double immunolabeled for Lmx1b and Phox2b. At this AP level, Lmx1b-expressing cells arise from the Phox2b-expressing pdB2 domain as well as the more ventral dB3 domain. (**L**) Cell counting of Phox2b^+^Lmx1b^+^-positive cells from the dB2 domain in r5 of control (n = 3) and Hoxa3^−/−^b3^−/−^ (n = 3) embryos, and r2/r3 of control embryos (n = 3). Because no Phox2b^+^Lmx1b^+^-positive cells were observed in the pdB2/dB2 domain of control embryos, the number of Phox2b^+^Lmx1b^+^-positive cells in r5 of Hoxa3^−/−^b3^−/−^ and r2/r3 of control embryos were naturally significantly more that r5 of control embryos. The bar represents standard deviation from the mean. Scale bars (A–C, E–K = 50 µm).
(TIF)
