# Supplementary figures and images for: Hoxb1 Controls Anteroposterior Identity of Vestibular Projection Neurons
Source: PLoS One. 2012 Apr 2;7(4):e34762. doi: 10.1371/journal.pone.0034762 (PMC3317634; doi:10.1371/journal.pone.0034762)

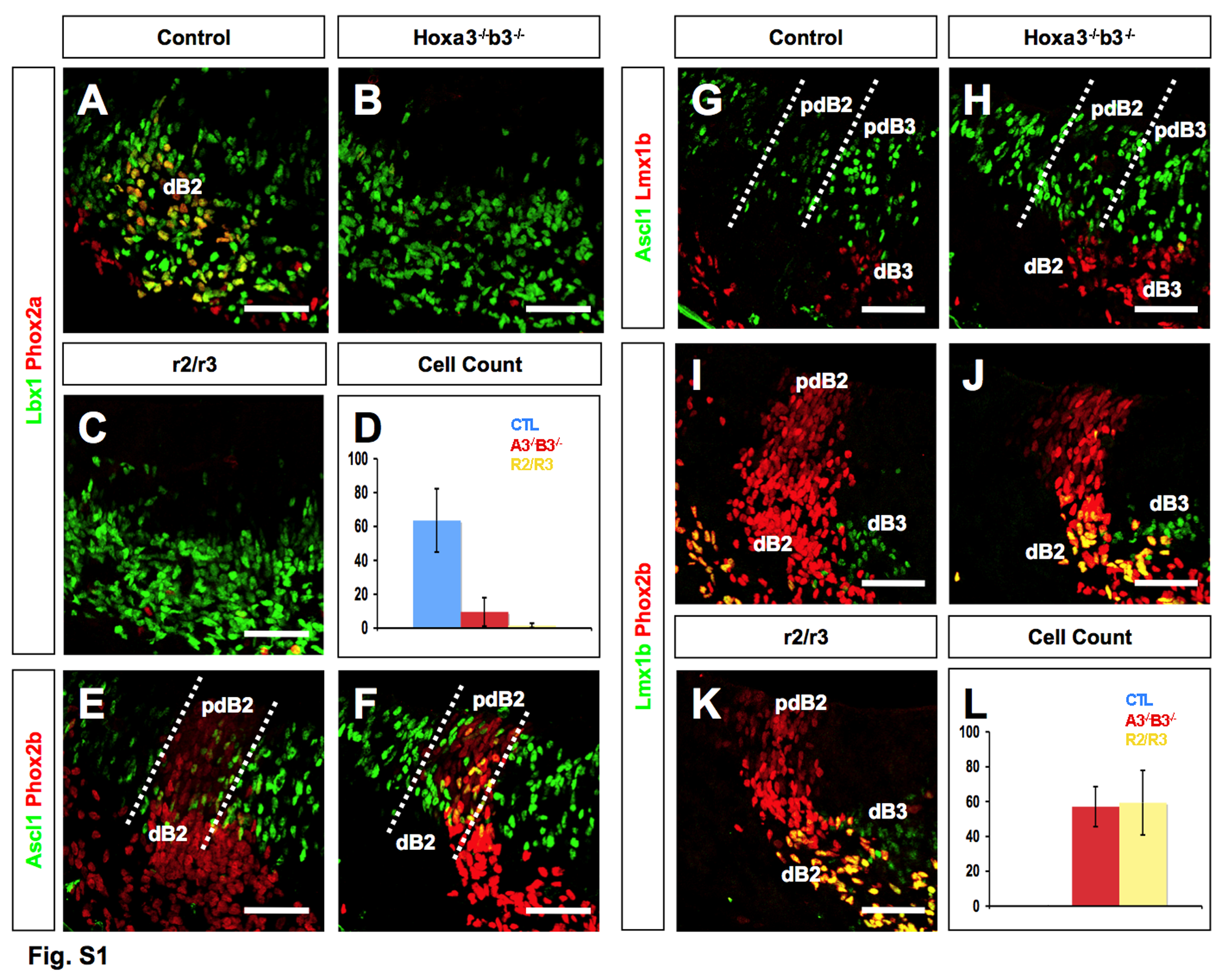

Supplement: Figure S1 — Hoxa3 and Hoxb3 are required for the AP identity of dB2 neurons in rhombomere 5. (A, B) Transverse sections through r5 of E11.5 control and Hoxa3−/−b3−/− embryos double immunolabeled for the HD proteins Phox2a and Lbx1. In the Hoxa3−/−b3−/− embryo, the expression of Lbx1 appears unperturbed whereas Phox2a is dramatically reduced. (C) Transverse sections through r2/r3 of E11.5 control embryo double immunolabeled for the HD proteins Phox2a and Lbx1. Phox2a at the intersection of this AP and DV level is expressed, if at all, at very low levels. (D) Cell counting of Phox2a+Lbx1+-positive cells in r5 of control (n = 3) and Hoxa3−/−b3−/− (n = 3) embryos, and r2/r3 of control embryos (n = 3). The number of Phox2a+Lbx1+-positive cells was significantly reduced in Hoxa3−/−b3−/− embryos compared to control embryos (P<0.05). Few Phox2a+Lbx1+-positive cells were found in r2/r3 of control embryos. The bar represents standard deviation from the mean. (E, F) Transverse sections through r5 of E11.5 control and Hoxa3−/−b3−/− embryos double immunolabeled for the bHLH transcription factor Ascl1 and Phox2b. The expression of Lbx1 appears unperturbed in the Hoxa3−/−b3−/− embryo, but Phox2a is dramatically reduced. Note that the expression of Ascl1 in the dB2 progenitor domain (pdB2) is relatively high in the Hoxa3−/−b3−/− embryo compared to the control. (G, H) Transverse sections through r5 of E11.5 control and Hoxa3−/−b3−/− embryos double immunolabeled for Ascl1 and Lmx1b. In the control embryo Lmx1b-expressing cells are associated with the dB3 progenitor domain (pdB3). In the Hoxa3−/−b3−/− embryo, Lmx1b-expressing cells ectopically associated with the pdB2 domain. (I, J) Transverse sections through r5 of E11.5 control and Hoxa3−/−b3−/− embryos double immunolabeled for the HD proteins Lmx1b and Phox2b. In the control, the expression of Lmxb1 in the dB3 domain is non-overlapping with the more dorsal Phox2b-expressing dB2 domain. In the Hoxa3−/−b3−/− embryo, the Lmx1b-expressing dB3 doma [file pone.0034762.s001.tif]
